# Round the Hospitals

**Published:** 1920-08-14

**Authors:** 


### ROUND THE HOSPITALS.

The King has awarded the Royal Red Cross, on the recommendation of the Government of India, to the following ladies in recognition of their valuable nursing services in the field in the Afghan War, 1919, to be dated January 1, 1920:—Royal Red Cross, Second Class.—*Queen Alexandra's Military Nursing Service for India*: The Misses F. B. Cholmondeley, matron (temp.); F. M. Clarke, nursing sister (temp.). *Temporary Nursing Service*: Miss A. Holmes, matron. *Australian Army Nursing Service*: Miss A. Hodson, sister. And in recognition of their valuable nursing services in connection with the Afghan War, 1919, to be dated January 1, 1920:—Royal Red Cross, First Class.—*Australian Army Nursing Service*: Miss G. Davis, principal matron. Second Class.—Miss C. Duncan, matron, Indian General Hospital. *Queen Alexandra's Imperial Nursing Service for India*:

**ROUND THE HOSPITALS**—(*continued*).

Miss M. Wardell, nursing sister. *Australian Army Nursing Service*: The Misses L. Campbell, matron; W. A. C. Gilliland, sister. In addition to the above, the names of the following ladies have been brought to notice for distinguished service during the operations against Afghanistan by General Sir C. C. Monro, G.C.B., G.C.S.I., G.C.M.G., in his despatch dated November 1, 1919, published in the Supplement of the *London Gazette* dated March 15:—*Queen Alexandra's Military Nursing Service for India*: Senior Nursing Sisters A. M. Gilmore, R.R.C., V. I. Lamb, R.R.C., and C. S. McGowan, R.R.C.; Nursing Sister M. D. Rabbidge; Temporary Nursing Sisters D. M. Scanlan and K. St. Martin. Also Temporary Nursing Sisters M. Mackintosh and E. O'Sullivan. *St. John Ambulance*: Temporary Nurse A. Higgins.

---

After six meetings held in private, the General Nursing Council admitted the Press to its meeting held at the Ministry of Health on July 30. The principal business transacted consisted in the recognition of the certificates of the Medico-Psychological Association, of the Fever Nurses' Association, and of the Metropolitan Asylums Board "as evidence of training and experience for admission of existing nurses to the Supplementary Parts of the Register for mental and for fever nurses respectively. This to apply to the period of grace only." The fee payable by nurses for admission to the register has been fixed at one guinea, with a further sum of half-a-guinea for admission to each and any further part of the Register. The names of Miss Cox-Davies, Miss Peterkin, Dr. Goodall, and Dr. Bedford Pierce have been added to the Education and Examination Committee. The question of "intermediate nurses" occupied the attention of the Council, and it was agreed that "nurses who produce a certificate of not less than three years' training from a general hospital or Poor-Law Infirmary recognised by the Council for training for the General Part of the Register, which has terminated at any period after November 1, 1919, but before the rules to be made by the Council for the education, examination, and training of nurses become operative, shall be admitted to the General Part of the Register." Intermediate nurses are to be admitted to the Male, Mental, and Children's Supplementary Parts of the Register on the same lines. With regard to intermediate fever nurses, the standard is fixed at two years' training and certification and one year's further service, as now in force.

---

We learn from the newly issued report of the treasurer of St. Thomas's Hospital for 1919 that Miss Lloyd Still's task in the reorganisation after the cessation of war conditions has been a particularly difficult one. "As Principal Matron of the 5th London General Hospital she was responsible for the demobilisation of the nursing unit of that hospital. All nurses other than V.A.D.s who had been employed there were Nightingale nurses. . . The St. John's and St. Thomas's House, which had been taken over in the previous year, was ready to absorb some of these nurses and has continued to do excellent work under the general supervision of the matron and the immediate charge of Miss Wilson, late night sister, and Miss Empson, formerly a sister of the hospital." The financial position causes much anxiety. The ordinary expenditure was £154,472, and the income from all sources only £111,954. Some of the increases since 1914 are enumerated. Salaries and wages were then just under £29,000. They rose to £39,596 in 1918, and last year totalled £50,763. The cost of provisions rose from £16,120 to close on £40,000. During the past year nurses' salaries have increased from £6,704 to £9,307. Great vigilance is exercised to ensure that all patients who can afford to do so pay a fair share of the expenses of treatment, and the lady almoners discuss with patients themselves how far they can contribute.

---

Thirty-two district nursing associations benefited by the recent distribution of the Metropolitan Hospital Sunday Fund; a sum of 2½ per cent. was set aside for these grants by the Committee. The grants varied according to the needs of the district and the work performed. The Ranyard Nurses come easily first with a grant of £750. Other associations receiving over £100 were East London, North London, Kensington, Paddington, and Marylebone, Plaistow, South London (Battersea), and Shoreditch. Nearly all the Metropolitan nursing associations are in great straits owing to increases in salaries, and it is abundantly clear that these have by no means reached their maximum.

---

The very existence of the Canadian Nurse-training schools is being threatened by the shortage of applicants. So we learn from the report of the President of the Canadian Association of Nursing Education quoted in the *Canadian Nurse* of July. To remedy this state of affairs Miss Flaws declares the schools must have adequate financial support, shorter hours, a reform which necessitates more probationers, better housing conditions, a reconstruction of the curriculum to allow students in their third year more time for administrative duties and public-health work, and a system of affiliation between small and large training-schools. It is thought that the Red Cross could help to bring the profession of nursing before the public and the high schools, and that every Red Cross member could become a missionary in spreading the gospel of nursing and interesting young women in the work. In England it seems pretty clear that this publicity department must be run by the hospitals themselves and that the Red Cross is ill-fitted to cater for the training-schools.

---

One of the reforms for which the Penal Reform League has been pressing most earnestly is the introduction of a regular staff of trained nurses into prison hospitals. The Home Secretary recently stated in the House of Commons that a trained hospital staff was in course of formation in Hollo-

**ROUND THE HOSPITALS—(*continued*).**

way Prison, consisting of a hospital lady superintendent, two principal nurses, and forty-eight nurses. We understand that Miss Jolley, R.R.C., the late matron of the Royal Air Force Nursing Service, and previously matron of the Royal Southern Hospital, Liverpool, is the lady superintendent of nursing. The details of training to be afforded have not yet been settled. It seems probable that they will embrace two distinct varieties of instruction: One, a short course to enable trained nurses, male and female, to become acquainted with prison rules and discipline, and enable the lady superintendent to form a judgment as to their fitness for nursing under prison conditions; the other, a First-Aid course for warders, or, as they are now termed, officials, to enable them to be of service, under trained nurses, in prison hospitals, and to increase their efficiency in the discharge of their ordinary duties. The advantage to prisoners, male and female, of being under the control of persons whose eyes have been opened by service under trained sisters is incalculable, and the wider the knowledge of elementary nursing ethics can be diffused in prisons the better.

---

If we were asked "When is a hospital not a hospital?" we should be tempted to answer, "When it has no nurses." Dr. Harold Balme, Dean of the Union Medical School at Tsinanfu, Shantung, has just published an "Inquiry into the Scientific Efficiency of Mission Hospitals in China." In order to obtain information, he circulated a *questionnaire* to 250 working hospitals, and he embodies the replies received in the above somewhat startling document. It appears that the conditions under which Medical Mission work is carried on in China leave very much indeed to be desired, and fully justify Dr. Balme in putting the question, "How far are our Mission hospitals able at the present time, in their attempt to represent and prosecute Christianity, to exhibit the highest ideals of careful and thorough medical and nursing work and thus avoid the belittling of that Christian message which it is their first purpose to propagate?" In the first place, 80 per cent. of the hospitals state they have only one doctor, trained in accordance with modern medicine and surgery—that is, outside China. It is quite obvious that the one-man hospital is a danger both to the missioner himself and his patients. Next we learn that 34 per cent. of the Mission hospitals in China have no nurses, either foreign or Chinese; 52 per cent. have no foreign (*i.e.*, trained) nurses; and 37 per cent. depend entirely on the patients' friends for all nursing. Moreover, 37 per cent. have no protection against flies or mosquitoes; 67 per cent. have no screening for their kitchens; 71 per cent. have no screening for their latrines. Do such "hospitals" serve any useful purpose?

---

The Queen has graciously consented to become Patroness of the Chartered Society of Massage and Medical Gymnastics, now beginning its new career, and it thus sets sail with a favouring breeze over the seas it has already, under different names, done much to sound and chart. The amalgamation of the Incorporated Society of Trained Masseuses with the Institute of Massage and Remedial Gymnastics has been safely accomplished, as we announced a short time ago, under the ægis of the Royal Charter, and already the results are apparent in the programme of the new Association. The Chartered Society is exceedingly fortunate in securing as Chairman of Council Sir E. Cooper Perry, consulting physician to Guy's Hospital and Principal Officer to London University. The most important immediate duty before the Society lies in setting up machinery for the registration of duly qualified members of the profession. The business of providing and supervising independent examinations, of still further improving the training and status of masseurs and masseuses, of arranging post-graduate courses, lectures, and conferences, of supplying work, under registry regulations, for its members, and of working for the general welfare of the profession will gain greatly in prestige by the possession of the Charter and the right of members to use the initials C.S.M.M.G. on cards and nameplates, though it may lose something in elasticity and freedom.

---

The Royal Devon and Exeter Hospital held an agreeable "At Home" on July 20, when the President, Brigadier-General Stirling, and Mrs. Stirling entertained 250 guests on the lawn. General Stirling made an earnest appeal for the building of the new wing, which is to cost £30,000, and to wipe out the adverse balance, which now stands at £4,500. He stated they were urgently in need of further accommodation for the nurses, and that it is intended to add an additional floor for this purpose to the new wing. They have only twelve nurses to send out to private cases. The President expressed the cordial thanks of the management to the matron, Miss Smale, R.R.C., and her staff, "on whom the success of the doctors depended."

---

It is very interesting to learn that the cost of provisions at Guy's Hospital in 1919 was no higher than in 1918. This experience we believe to be unique. Not only did prices go on increasing steadily during the whole year, but the imperative need for economy was relaxed by the withdrawal of the rationing orders. The Treasurer reports that this remarkable achievement is due to the elimination of waste, and ascribes it largely to the efforts of the sisters of the wards. Unavoidable increases have, of course, taken place in many other directions, but the cost of the laundry has actually not risen, and the price per piece stands at the astonishing figure of 1.27 pence, or about 1¼d.

---

Southwark Borough Council has been recommended by its Maternity and Child Welfare Committee to appoint six additional health visitors. The Ministry of Health has reduced the number of

**ROUND THE HOSPITALS—***(continued)*.

births that one visitor may attend to from 400 to 250 a year. At some of the centres as many as 400, 500, and 600 births are added annually to the register of infants to be attended to, and unless the six extra visitors are provided many of these infants may be left unvisited. When the scheme was first inaugurated there was an outcry in many districts that there was too much interference in home life, and that mothers would resent it. But this fear has not been justified, and, with very few exceptions, mothers gladly welcome the health visitor.

---

A strenuous struggle is being fought in Lambeth to place the salaries of two tuberculosis nurses employed by the Borough Council on the same level as the salaries of the lady health visitors and sanitary inspectors. Some time ago the Council expressed the opinion that the tuberculosis nurses should be placed on exactly the same footing as the other officers with respect to salary, having regard to their qualifications and duties. Later the Council placed its women inspectors and health visitors on a post-war basis with respect to salary, but unfortunately the tuberculosis nurses were accidentally omitted. Several attempts have been made to remedy this omission, but hitherto without success, the General Purposes Committee on two occasions declining to give the tuberculosis nurses the same salaries as the health visitors and sanitary inspectors. The salary of the tuberculosis nurses at present are £316 2s. 6d. a year, including war bonus, but if the Council did justice to them they would receive £356 and £350 respectively. The Council is almost equally divided on the matter, for when at the last meeting a resolution was adopted that the committee should bring up a resolution to adjust the salaries of the tuberculosis nurses it was only carried by the narrow majority of one vote, twenty-six councillors being in favour of equality and twenty-five against.

---

In reply to queries relating to ration allowances, we learn that the custom varies considerably in different institutions. At the Royal Victoria Infirmary, Newcastle-on-Tyne, no ration allowance is paid to members of the nursing staff when on leave, and we believe this to be the practice in all voluntary hospitals. At the Crumpsall Infirmary, Manchester, nurses are allowed 15s. weekly for board during their annual holiday, but no ration allowance for their odd days off. Always, however, a nurse may take all her meals in the hospital on these occasions should she desire to do so. Under the Metropolitan Asylums Board nurses and other employees who are in receipt of a mixed remuneration of cash and emoluments are all granted allowance in lieu of board when on occasional leave. They are thus treated in the same way as members of the staff who are on inclusive cash salaries; that is, they do not pay for board of which they do not partake. It will be seen that the position can be regarded from more than one point of view. The hospitals which do not give ration allowances engage their nurses at a salary which includes board during the time that they are on duty but not during the annual holidays or on such days and half-days as they may choose to spend away from the institution.

---

The Minister of Health states that nurses and other officers of Boards of Guardians are not entitled as a matter of course to money allowances in lieu of rations or part rations while on leave, unless this had definitely been made a part of the terms of appointment. "It is, however, the practice of the Minister to sanction such allowances in any case in which sanction is necessary if the Guardians so desire, the arrangement being regarded as, in effect, an increase of the remuneration of the officer." This is a matter of considerable moment to nurses at the present time, when the privilege of getting away once a week is considerably curtailed by the cost of rations and annual holidays make terrible demands on the exchequer.

---

Wandsworth Borough Council is anxious to get on with the scheme for a maternity home at Park Hill, Tooting, an enormous house with thirty-five rooms and standing in grounds of three acres. The freehold of the place costs £5,250, and plans for the necessary alterations have been approved by the Ministry of Health. There will be provision for twenty-four beds and the necessary special wards, accommodation for staff, and a laundry. The cost of adaptation and equipment is estimated at £5,800. It is proposed to make a charge to patients based on a sliding scale, patients paying according to their income, and ranging from a charge of 15s. a week in the public wards to a maximum of four guineas a week in the private wards. Any patient will be allowed to have her own medical adviser, if preferred, at her own expense. Cost of maintenance is expected to be £4,450 per annum, and the estimated receipts about £1,200, leaving a balance of about £3,250, of which fifty per cent. will be paid by the Ministry of Health.

---

The little accessories of the table which add so much to the meal are prohibitive in cost at the present time and some of our readers may have been reduced to cutting down salad dressings and sauces altogether. The following is an inexpensive preparation which may be used as a substitute for salad dressing and will be found to possess exactly the right flavour. Ingredients: salt, one tablespoon; mustard, three large teaspoons; sugar, two tablespoons; cayenne, a good dash; flour, one-half tablespoon, or more if required thick; butter, size of an egg; milk, two large cups; vinegar three-quarter cup. Mix the dry ingredients very thoroughly in saucepan until quite smooth; add milk gradually and vinegar last. Boil for three minutes. This is excellent with cold meat with or without salad.

